# First-Principles Study on Stability and Magnetism of Ni_*m*_Al_*n*_ (*m* = *1*–*3*, *n* = *1*–*9*) Clusters

**DOI:** 10.1155/2013/468327

**Published:** 2013-05-24

**Authors:** Xiao Zhang, Bao-Xing Li, Zhi-wei Ma, Jiao-jiao Gu

**Affiliations:** Department of Physics, Hangzhou Normal University, Hangzhou, Zhejiang 310036, China

## Abstract

The investigation on the structures, stabilities, and magnetism of Ni_*m*_Al_*n*_ (*m* = 1–3, *n* = 1–9) clusters has been made by using first principles. We found some new ground-state structures which had not been found before. These mixed species prefer to adopt three-dimensional (3D) structures starting from four atoms. All the ground-state structures for the Ni-Al clusters are different from those of the corresponding pure Al clusters with the same number of atoms except for three atoms. The Mulliken population analysis shows that some charges transfer from the Al atoms to the Ni atoms. NiAl_*n*_ (*n* = odd number) cations, Ni_2_Al_6_ neutral, Ni_2_Al_1_ and Ni_3_Al cations and anions, and Ni_3_Al_5_ anion have the magnetic moments of 2 *μ*_B_. The magnetic moments of NiAl_4_ and NiAl_6_ cluster neutrals and cations are 2 *μ*_B_ and 3 *μ*_B_, respectively. All the other cluster neutrals and ions do not have any nontrivial magnetic moments. The 3d electrons in Ni atoms are mainly responsible for the magnetism of the mixed Ni-Al clusters.

## 1. Introduction

Aluminum is one of the most popular metals used in quantum-effect electronic devices. It attracts scientists' great attention to its nanoscale clusters. Cohen et al. have explained the stability of small Al_*n*_ clusters by using jellium model because of the valence electrons of Al-like free electron [1, 2]. Khanna et al. have discovered that aluminum clusters have chemical properties similar to single atoms of metallic and nonmetallic elements when they react with iodine [3, 4]. For instance, Al_13_ cluster behaves like a single iodine atom, while Al_14_ cluster is similar to an alkaline earth atom. Fournier have obtained the ground-state structures of Al_*n*_, Al_*n*_^−^,   and  Al_*n*_^+^  (4 ≤ *n* ≤ 15) by performing “Tabu Search” (TS) global optimizations directly on the BPW91/LANL2DZ potential energy surface [5]. They have found that all clusters (4 ≤ *n* ≤ 15) have the lowest spin state as their ground state except Al_4_ (triplet), Al_4_^+^ (quartet), Al_7_^−^ (triplet), and maybe Al_5_^+^ (singlet and triplet are degenerate). Furthermore, Al_7_^+^ and Al_13_^−^ had special stability and larger HOMO-LUMO gaps.

The studies of the Al clusters doped with impurity atoms have been also reported. Ren and Li have investigated the structures, stabilities, and magnetism of zinc-doped Al_*n*_ (*n* = 1–9) clusters in detail by using first-principles density functional theory [6]. Ding and Li have done the calculation on Al_*n*_Si_*m*−*n*_  (*m* = 6, 9,  10,  *n* ≤ *m*) by using the first-principles methods [7]. Li and Wang have performed first-principles calculations on the ground states of both neutral and anionic Al_12_ *X*  (*X* = C, Si, Ge, Sn, Pb) clusters [8].

Nickel is a transition metal. Aluminum clusters doped Ni atoms have many novel properties and chemical properties. Ni_*n*_Al  (*n* = 2–8) neutral clusters have been investigated by Wen et al. using the density functional theory and generalized gradient approximation (GGA) with the exchange-correlation potential (BPW91) [9]. It is found that the atomization energies per atom have the same trend as the binding energies per atom for Ni_*n*_  (*n* = 3–9) clusters. It is shown that Ni_5_Al is the relatively most stable structure in the series. Besides that,  Ni average magnetic moment decreases when alloyed with Al atoms than that in pure Ni clusters. Deshpande et al. have reported the magnetic properties of small Ni_13−*n*_Al_*n*_ clusters with *n* = 0–13 calculated in the framework of density functional theory [10]. The overall magnetic moment of the Ni_13−*n*_Al_*n*_ cluster decreases with the sequential substitution of Ni by Al atoms. This could be attributed to the antiferromagnetic alignment of small individual atomic moments arising due to the hybridization of Al-p and Ni-3d orbitals. Calleja et al. have reported ab initio molecular dynamics simulations of Ni_2_,  Al_2_,  Ni_13_,  Al_13_,  and  Ni_12_Al clusters using a fully self-consistent density-functional method [11]. Recently, Sun et al. have performed the study on the growth behavior and electronic properties of NiAl_*n*_  (*n* = 1–14) clusters in the framework of density-functional theory (DFT) theoretically [12]. In the ground-state structures of NiAl_*n*_ clusters, the equilibrium site of Ni atom gradually moves from convex and surface to interior site as the number of Al atom varying from 2 to 14. Ferrando et al. have performed very thorough review on alloyed clusters [13]. Bailey et al. have presented their research results about nickel and aluminum clusters and nickel-aluminum nanoalloy clusters with up to 55 atoms [14]. The effect of doping Al atoms into pure Ni clusters and vice versa has been investigated.

Although the investigations about Al, Ni, and Ni_*n*_Al clusters mentioned above have been performed, our knowledge about the properties of the Ni-Al clusters with different Ni/Al atomic ratio is still limited. In this paper, our main goal is to explore the effect of the Ni atoms on the geometric, electronic, and magnetic properties of the pure Al_*n*_  (*n* = 2–9) clusters. 

The paper is organized as follows: a brief account of the computational methodology is given in Section 2, followed by a detailed presentation and discussion of the structures of different size Ni_*m*_Al_*n*_  (*n* = 1–9, *m* = 1–3) clusters in Section 3. A summary of the findings and conclusions is given in Section 4.

## 2. Computational Method

We optimized the Ni-Al cluster structures by using first-principles density functional theory. Our calculations were performed with the generalized gradient approximation (GGA) by means of the Becke-Perdew functional, which uses Becke's [15] gradient correction to the local expression for the exchange energy and Perdew's [16] gradient correction to the local expression of the correlation energy, as implemented in the Amsterdam Density Functional (ADF) codes [17]. The choice of such a gradient-corrected exchange-energy functional gives us satisfactory results, which are in good agreement with the experimental results. For example, the calculated vertical electron affinity and vertical ionization potential for Al_5_ cluster are 2.16 eV and 6.61 eV, respectively. The available experimental values are 2.25 eV and 6.45 eV, respectively [18, 19]. In the ADF program, molecular orbitals (MOs) were expanded using a large, uncontracted set of Slater-type orbitals (STOs): TZ2P. The TZ2P basis is an all-electron basis of triple-*ζ* quality, augmented by two sets of polarization functions. The frozen-core approximation for the inner-core electrons was used. The orbitals up to 3p  for nickel and 2p for aluminum were kept frozen. An auxiliary set of *s*, *p*, *d*, *f*, and  *g* STOs was used to fit the molecular density and to represent the Coulomb and exchange potentials accurately in each self-consistent field (SCF) cycle. The self-consistent field was converged to a value of 10^−5^ in Hartree. 

Frequency analyses at the stable structures are carried out at the same theoretical level to clarify if they are true minima or transition states on the potential energy surfaces of specific clusters. All of the most stable clusters in the paper are characterized as energy minima without imaginary frequencies. 

## 3. Results and Discussions

Searching for the ground-state structures of the atomic clusters, especially the mixed clusters, is a difficult task. The following approach is applied in an attempt to find the global energy minimum structures. Firstly, about more than ten thousand initial geometrical configurations are produced by random selections of atomic positions within a three-dimensional box, or a cage, or a ball in real space. The distance between two nearest atoms is properly chosen to avoid overlapping or loosely packing. Next, the Amsterdam Density Functional (ADF) package is used for geometrical optimizations with their spins being not unrestricted. After the structural optimization on the initial configurations is performed, it is found that some structures are stable, but others are not convergent. Sometimes, several initial structures can gave out the same final structure after the first-principles optimizations. In the end, the structures with the largest binding energies calculated using generalized gradient approximation (GGA) are considered to be the ground-state geometries. The binding energy (BE) for the Ni_*m*_Al_*n*_ cluster is calculated according to the following atomization reaction: Ni_*m*_A1_*n*_ → *m*Ni + *n*A1. It is defined by the following: BE = *mE*_Ni_ + *nE*_A1_ − *E*_Ni_*m*_A1_*n*__, where *E*_Ni_*m*_A1_*n*__ is the total energy of the Ni_*m*_Al_*n*_ cluster. *E*_Ni_ and *E*_Al_ are the energy of Ni and Al atoms, respectively. 

**Figure 1:**
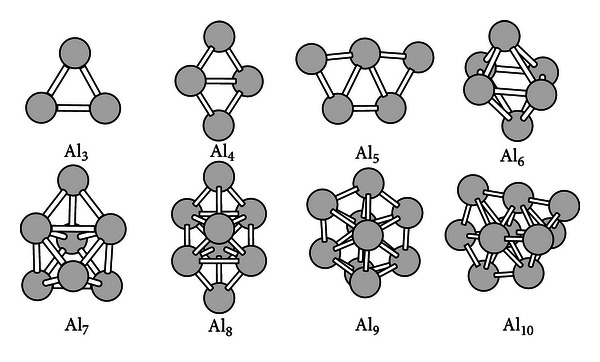
The ground-state structures of the Al_*n*_  (*n* = 3–10) clusters.

All the lowest energy structures of Al_3_, Al_4_, and Al_5_ clusters are planar structures. They are an equilateral triangle, a planar quadrangle, a trapezoid-like, respectively. The ground-state structures for Al_*n*_  (*n* = 6–10) clusters are three-dimensional. The Al_6_ and Al_7_ clusters have a distorted tetragonal bipyramid (D_2d_) and an antitrigonal prism with a capping atom (C_3v_), respectively. But, Fournier found that the ground-state structure for the Al_6_ cluster was a distorted trigonal prism by using the BPW91/LANL2DZ method [5]. For the Al_7_ cluster, its ground-state structure can be obtained by putting an atom on one triangular face of the prism for the Al_6_ cluster. We have also performed calculations on the two structures found by Fournier with the ADF program. It is found that both of them are metastable. Our results are 0.15 and 0.36 eV more stable than his, respectively. For the Al_8_ cluster, our result is a transcapped octahedron with C_2 h_ symmetry. But, in Fournier's report, the ground-state structure is a distorted transcapped octahedron, or an antitrigonal prism with a face-capping atom and an edge-capping atom. We have performed structural optimization on his structure. It is found that his structure finally changes into the C_2h_ structure. The Al_9_ cluster has a C_2v_ ground-state structure. The most stable structure of pure Al_10_ cluster can be obtained from the Al_9_ ground-state isomer by putting a capping atom. All of the structures obtained by the ADF program are in excellent agreement with those reported by Chuang et al. using a genetic algorithm coupled with a tight-binding interatomic potential [20]. They are shown in Figure 1.

**Figure 2:**
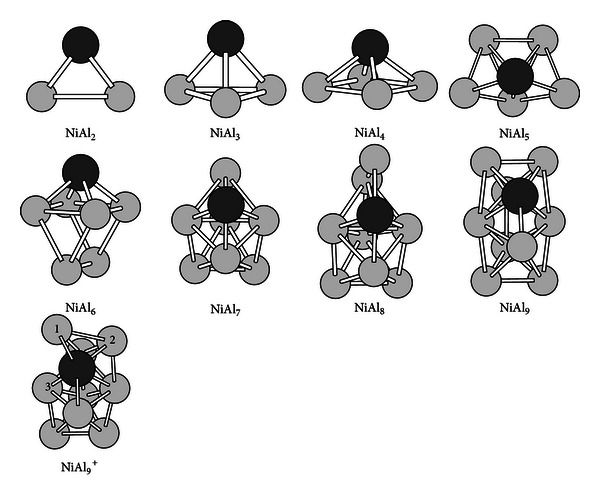
The ground-state structures of the NiAl_*n*_  (*n* = 2–9) clusters. The black ball refers to Ni atom in the clusters.

The ground-state structures of NiAl_*n*_  (*n* = 2–9) clusters are presented in Figure 2. The ionic structures similar to the neutral structures are not repeated in Figure 2. But the structure of NiAl_9_ cluster cation is presented in Figure 2 because it has a severe distorted ground-state structure compared with its neutral and anion. Their symmetries are listed in Tables 1, 2, and 3. The NiAl_3_ and NiAl_4_ clusters and their ions have one three-dimensional structure, which are different from the planar structures of the Al_4_ and Al_5_ clusters. All the most stable structures for the NiAl_*n*_  (*n* = 5–9) cluster neutrals, anions and cations are three-dimensional structures, but they are obviously different from the structures of the corresponding Al_*n*_  (*n* = 6–10) clusters with the same number of atoms. Positive NiAl_9_ cluster ion with C_1_ symmetry has a severe distorted ground-state structure compared with its neutral and anion (see the NiAl_9_^+^ structure in Figure 2). Mulliken population analysis shows that nonuniform charge loss of the Al atoms in the NiAl_9_^+^ structure results in its severe structure distortion.

The cluster's magnetism is usually described by its magnetic moment. We define magnetic moment (in Bohr magnetron unit *μ*_B_) as the difference between the electronic occupation number of spin-up and spin-down. We think the cluster is magnetic if its structure with nonzero spin is more stable than that with zero spin. The investigation on the magnetic moments for the Ni-Al clusters shows that anions, neutrals, and cations for the NiAl_2_ and NiAl_8_ clusters have the magnetic moments of 1 *μ*_B_, 0, 1 *μ*_B_, respectively. Negative, neutral, and positive NiAl_4_ and NiAl_6_ clusters have the magnetic moments of 1 *μ*_B_, 2 *μ*_B_, and 3 *μ*_B_, respectively. The NiAl,  NiAl_3_,  NiAl_5_,  NiAl_7_,  NiAl_9_ clusters with odd-aluminum atoms have the magnetic moments of 1 *μ*_B_. Their negative ions have no magnetic moment, but the positive ions show the magnetic moment of 2 *μ*_B_. 

**Figure 3:**
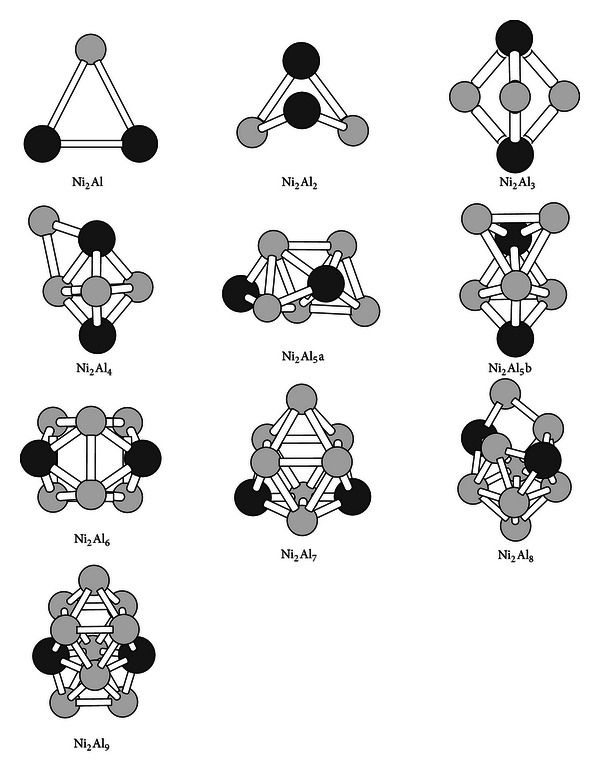
The ground-state structures of the Ni_2_Al_*n*_  (*n* = 2–9) clusters. The black ball refers to Ni atoms in the clusters.

The most stable structures of Ni_2_Al_*n*_  (*n* = 1–9) clusters are presented in Figure 3. It is found from observing Figure 3 that the geometries are also different from those in Figures 1 and 2. Obviously, the second impurity Ni atom further changes the structures of Al host clusters. The lowest energy structure Ni_2_Al_5_a of Ni_2_Al_5_ cluster which is obtained by a random way has a C_1_ symmetry. The second most stable structure Ni_2_Al_5_b which produced by substitution from Al_8_ has a C_s_ symmetry and lies 0.07 eV above Ni_2_Al_5_a structure. All the ground state structures of the Ni_2_Al_6_, Ni_2_Al_7_,  and  Ni_2_Al_9_ clusters have the same C_2v_ symmetry. The Ni_2_Al_7_ cluster can be obtained by substitution, by a random way, or by putting a capping atom on the Ni_2_Al_6_ cluster. The Ni_2_Al_8_ cluster has a low C_1_ symmetry.

By performing calculation on the magnetic moments for the Ni_2_Al_*n*_  (*n* = 1–9) clusters, we obtain the following results. All the Ni_2_Al_*n*_  (*n* = odd-number) clusters have the total magnetic moment of 1.0 *μ*_B_ due to one unpaired electron. This suggests that the two impurity Ni atoms do not change the magnetism of the Al clusters. On the other hand, the Ni_2_Al_2_, Ni_2_Al_4_, and Ni_2_Al_8_ clusters with even number of electrons have no magnetic moment because all the electrons are paired together in their respective molecular orbitals. But, unexpectedly, the Ni_2_Al_6_ cluster shows magnetic moment of 2 *μ*_B_. Each Ni atom in the Ni_2_Al_6_ cluster has the local magnetic moment 0.40 *μ*_B_ and the total charge transferring from the Al atoms to them is −0.74 e. However, the average magnetic moment of each Al atom is 0.20 *μ*_B_, which is only half of that of the Ni atoms. The Mulliken population analyses indicate that the local magnetic moment 0.40 *μ*_B_ of Ni atoms is mainly from the d orbital. On the other hand, if we add up the magnetic moments for the Al atoms and the Ni atoms, respectively, the total magnetic moments for Al atoms are 1.2 *μ*_B_, which is larger than 0.8 *μ*_B_ for Ni atoms. Hence, Al atoms contribute more to total spin than Ni atoms. 

The Ni_2_Al^−^  and  Ni_2_Al^+^ cluster ions are expected to produce zero magnetic moment. But our calculations show that the ions have the magnetic moment of 2 *μ*_B_. It is found that the Ni_2_Al_3_,  Ni_2_Al_5_,  Ni_2_Al_7_, and  Ni_2_Al_9_ clusters ions have no magnetic moment. The magnetic moment for the Ni_2_Al_4_,  Ni_2_Al_6_,  and  Ni_2_Al_8_ cluster ions is 1 *μ*_B_. Furthermore, it is found that the electron added or removed in the neutral Ni_2_Al_*n*_  (*n* = 1–9) clusters does not change the basic geometrical structures. 

**Figure 4:**
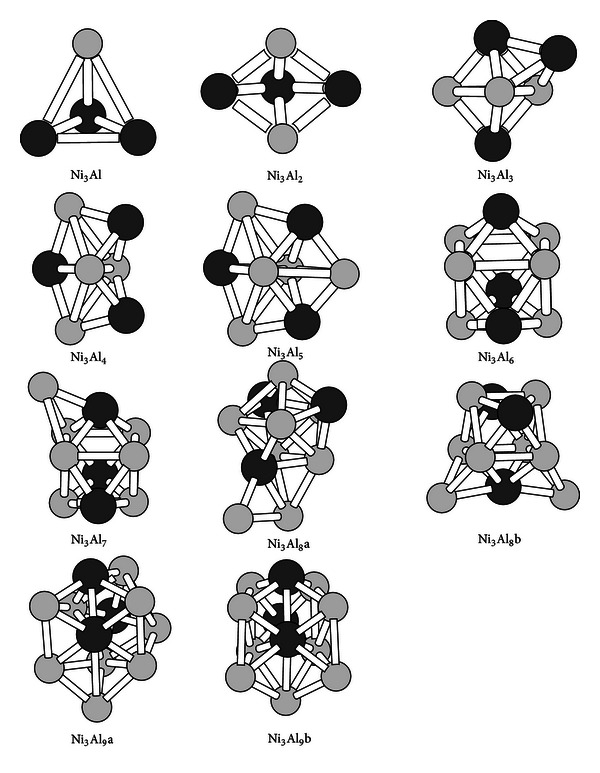
The ground-state structures of the Ni_3_Al_*n*_  (*n* = 2–9) clusters. The black ball refers to Ni atoms in the clusters.

The lowest energy structures of Ni_3_Al_*n*_  (*n* = 1–9) clusters are presented in Figure 4. All the structures are three-dimensional. They differ from the geometries of the NiAl_*n*_ and Ni_2_Al_*n*_  (*n* = 1–9) clusters in Figures 2 and 3. The clusters have C_3v_, C_2v_, C_s_, or  C_1_ symmetry. The ground-state structure of the Ni_3_Al_8_ cluster is degenerate. The Ni_3_Al_8_a structure lies only 0.03 eV below the Ni_3_Al_8_b structure. The Ni_3_Al_8_b structure can be obtained by putting two Al capping atoms in the Ni_3_Al_6_ structure. The Ni_3_Al_9_a structure is 0.26 eV more stable than the Ni_3_Al_9_b structure. 

The Ni_3_Al_*n*_  (*n* = odd-number) clusters have the magnetic moment of 1.0 *μ*_B_ due to one unpaired single electron. The magnetic moment is trivial. For the Ni_3_Al_*n*_  (*n* = even-number) clusters show no magnetic moment because the molecular orbitals are doubly occupied. For their ions, some clusters show magnetism. The Ni_3_Al^−^, Ni_3_Al^+^, and  Ni_3_Al_5_^−^ cluster ions are expected to produce zero magnetic moment. But our calculations indicate that they have the magnetic moment of 2 *μ*_B_. All the Ni_3_Al_3_, Ni_3_Al_7_, and Ni_3_Al_9_ clusters ions including the Ni_3_Al_5_^+^ cation have no magnetic moments. The magnetic moment for the Ni_3_Al_4_, Ni_3_Al_6_, and Ni_3_Al_8_ clusters ions is 1 *μ*_B_, which is trivial.

**Figure 5:**
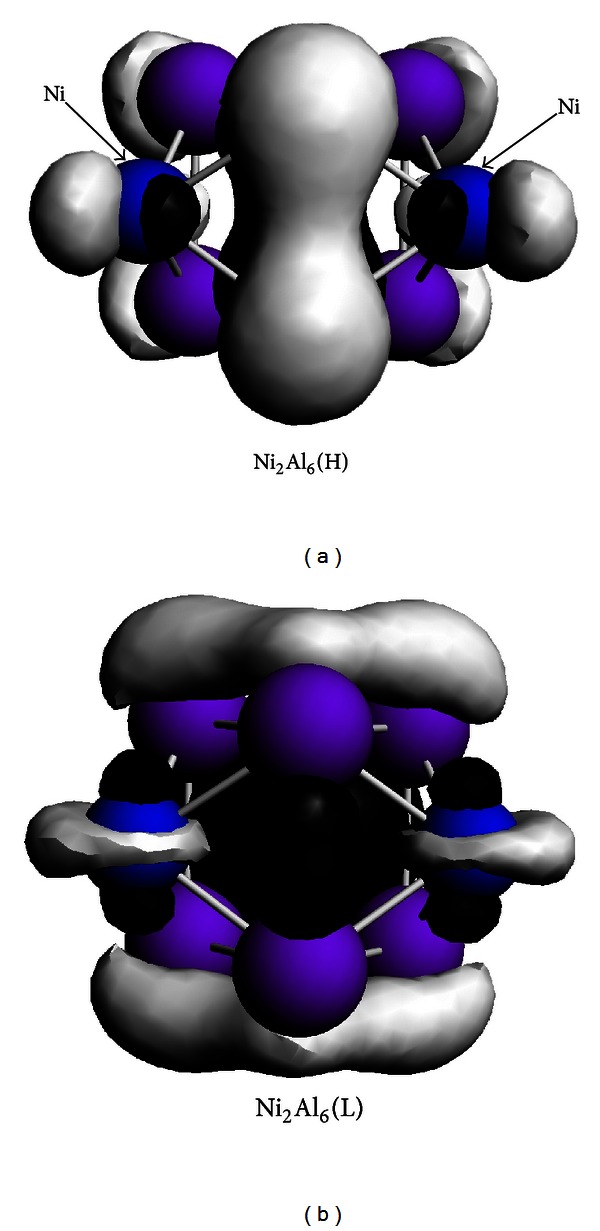
The HOMO and LUMO orbitals of the Ni_2_Al_6_ cluster.

To investigate the bonding character of the mixed Ni-Al clusters in relation to molecular orbitals, we have also examined the density of states in terms of the contributions of the different orbital components (s, p, d) and the frontier orbital (HOMO and LUMO) states. The Mulliken population analyses indicate that some of charge transfer from the Al atoms to the Ni atoms. Here, we take the Ni_2_Al_6_ cluster with the magnetic moment of 2 *μ*_B_ as an example to illustrate their bonding properties. The orbitals of HOMO and LUMO states of the Ni_2_Al_6_ cluster are shown in Figure 5. For the orbitals of the HOMO state, the cluster binds in the term of *σ* and *π* orbital. Likewise, the binging characteristic varies from *σ* orbital to *π* orbital and *δ* feature between Ni and Al atoms for the LUMO state. 

**Figure 6:**
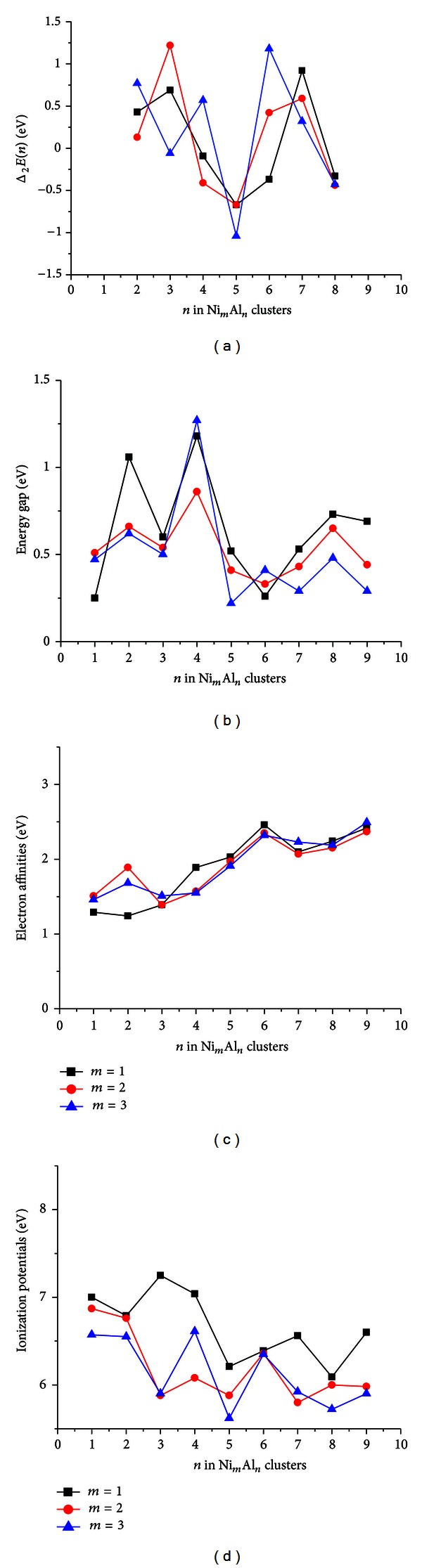
The second difference of the cluster energies, the energy gaps *E*_*g*_ between the highest-occupied molecular orbital (HOMO) and the lowest-unoccupied molecular orbital (LUMO), the electron affinities (EA), and the ionization potentials (IP) for Ni_*m*_Al_*n*_  (*m* = 1–3, *n* = 1–9) clusters.

In order to better understand the relative stability of the Ni-Al clusters, we have also investigated the second difference of cluster energies, the energy gaps, and their positive and negative ion clusters. The second difference of cluster energies Δ_2_ *E* = *E*(Ni_*m*_Al_*n*+1_) + *E*(Ni_*m*_Al_*n*−1_) − 2*E*(Ni_*m*_Al_*n*_)  (*m* = 1–3, *n* = 2–9), which is a sensitive quantity that reflects the relative stability of the mixed clusters. Maximum Δ_2_ *E* indicates that the cluster is more stable than its neighboring clusters. The maximums for the Ni_1_Al_*n*_  (*n* = 1–9)  and  Ni_2_Al_*n*_  (*n* = 1–9) clusters are found at *n* = 3  and  7 (see Figure 6). The conclusion is a good agreement with that obtained by Sun et al. [12]. For the Ni_3_Al_*n*_  (*n* = 1–9) clusters, the maximums are at *n* = 4  and  6. It is found from observing Figure 6 that the minimum for the mixed clusters is at *n* = 5. This implies that the Ni_*m*_Al_5_  (*m* = 1–3) clusters are less stable than their neighboring clusters. 

The maximums of Δ_2_E for the pure Al_*n*_  (*n* = 2–10) clusters are also at *n* = 3  and  7. The stability probably can be explained by jellium model [2, 21]. In this model, the nuclei together with the innermost electrons form a positive charged background, whereupon the valence electrons coming from individual atoms are then subjected to this potential. The clusters containing 8, 20, 40, 58… valence electrons correspond to filled electronic shells and exhibit enhanced stability. But the stability is associated not only with closed electronic shells (*Ne* = 20, 40) but also, to a lesser extent, with numbers of electrons just above and below the shell closing numbers [5]. The simultaneous stability of the neutral, anion, and cation of a given size can happen only for clusters of elements having more than one itinerant electron per atom. Therefore, the higher stability of Al_3_  and  Al_7_ clusters is easily understood. It is found from observing Figure 6 that the mixed Ni_*m*_Al_3_  and  Ni_*m*_Al_7_  (*m* = 1  or  2) clusters also have high stability. This indicates that the stability of the nickel-doped Al_*n*_ clusters is determined by the host Al clusters. But it should be noted that more heteroatoms would change their relative stability, as we have seen in the Ni_3_Al_*n*_  (*n* = 2–9) clusters. Al_5_ cluster is less stable compared to other Al clusters. This is the reason that the Ni_*m*_Al_5_  (*m* = 1–3) clusters are less stable than their neighboring clusters. 

The energy gaps *E*_*g*_s as an important function of the cluster size present a characteristic quantity of metal clusters' chemical activity. Here we define *E*_*g*_s as the gap between the highest occupied molecular orbital (HOMO) and the lowest unoccupied molecular orbital (LUMO). For the pure Al_*n*_ clusters, it is found that the Al_2_ cluster has a large gap of 3.05 eV. Other gaps change within range from 0.32 to 0.84 eV. For the smaller mixed clusters, their energy gaps change drastically. But they show relatively small fluctuation as the atomic number increases. It is worth noting that the *E*_*g*_ curves display an even/odd alternating pattern as a function of cluster size for the Ni_3_Al_*n*_  (*n* = 1–9) clusters. Even though the Ni_1_Al_*n*_  and  Ni_2_Al_*n*_  (*n* = 1–9) clusters with larger than five atoms do not present the even/odd alternating pattern, their change trend is similar as the atomic number increases. 

Finally, we have also investigated the Ni_*m*_Al_*n*_  (*m* = 1–3, *n* = 1–9) positive and negative ion clusters. The electron affinities EA = *E*(Ni_*m*_Al_*n*_) − *E*(Ni_*m*_Al_*n*_^−^)  (*m* = 1–3, *n* = 1–9) are the amount of energy released when the cluster obtains an electron from its neutral state. In our research, all the vertical electron affinities (EA) of the Ni_*m*_Al_*n*_  (*m* = 1–3, *n* = 1–9) clusters are positive, suggesting that the clusters have a tendency to gain an electron, as it is energetically favorable to do so. The vertical electron affinities (EA) change with atomic number, shown as Figure 6. The graph of vertical electron affinities versus atomic number shows that the mixed clusters with one, two, and three nickel atoms have a similar change trend. For the positive ion clusters, similar energies called vertical ionization potentials are calculated. The graph of vertical ionization potentials versus atomic number displays different changes. For the Ni_2_Al_*n*_  (*n* = 2–9) clusters with two nickel atoms, the vertical ionization potentials (IP) show an even/odd alteration with the number of aluminum atoms. For the Ni_3_Al_*n*_  (*n* = 2–9) clusters with three nickel atoms, a similar change trend (except  for  *n* = 8) is observed. But, for the mixed clusters including one nickel atom, the property does not exist. 

## 4. Conclusions

We have optimized the geometric structures of the mixed Ni_*m*_Al_*n*_  (*m* = 1–3, *n* = 1–9) clusters by using first-principles density functional theory. Their ground-state structures are obtained. Most of them are different from those of the host Al clusters. The impurity Ni atoms result in the local structural distortion of the mixed clusters in comparison with the corresponding pure Al clusters. At the same time, we calculated magnetic moment of the mixed cluster neutrals and ions. Some of them present nontrivial magnetic moments. The NiAl_*n*_  (*n* = odd  number) positive ions, the Ni_2_Al_6_  neutral, the  Ni_2_Al_1_  and  Ni_3_Al positive and negative ions, and the Ni_3_Al_5_ negative ion possess the magnetic moments of 2 *μ*_B_. NiAl_4_  and  NiAl_6_ cluster neutrals and positive ions have the magnetic moments of 2 *μ*_B_ and 3 *μ*_B_, respectively. But all the other cluster neutrals and ions do not have any nontrivial magnetic moments. Some of charge transfer from the Al atoms to the Ni atoms. The local magnetic moment of Ni atoms is mainly from the d orbital. Both the structural effect and chemical bonding are responsible for the magnetism of the mixed Ni-Al clusters.

## Figures and Tables

**Table 1:** The symmetries, the total magnetic moments (*M*, in *μ*_B_), and the binding energies (BE, in eV) for NiAl_*n*_ (*n* = 2–9) clusters and their ions. The energy gaps (*E*_*g*_, in eV), the vertical electron affinities (EA, in eV), and vertical ionization potentials (IP, in eV) of neutral NiAl_*n*_ (*n* = 2–9) clusters.

Clusters	Symmetries	*M* (in *μ*_B_)			BE (in eV)			*E*_*g*_ (in eV)	EA (in eV)	IP (in eV)
		Anion	Neutral	Cation	Anion	Neutral	Cation			
NiAl	C_*∞*v_	0	1	2	4.82	3.53	3.47	0.25	1.29	7.00
NiAl_2_	C_2v_	1	0	1	8.18	6.94	0.15	1.06	1.24	6.79
NiAl_3_	C_3v_	0	1	2	11.31	9.92	2.67	0.60	1.39	7.25
NiAl_4_	C_4v_	1	2	3	14.10	12.21	5.17	1.18	1.89	7.04
NiAl_5_	C_s_	0	1	2	16.62	14.59	8.38	0.52	2.03	6.21
NiAl_6_	C_2v_	1	2	3	20.10	17.64	11.25	0.26	2.46	6.39
NiAl_7_	C_s_	0	1	2	23.16	21.06	14.50	0.53	2.10	6.56
NiAl_8_	C_s_	1	0	1	25.80	23.56	17.47	0.73	2.24	6.09
NiAl_9_	C_s_	0	1	2	28.81	26.39	19.99	0.69	2.42	6.60

**Table 2:** The symmetries, the total magnetic moments (*M*, in *μ*_B_), and the binding energies (BE, in eV) for Ni_2_Al_*n*_ (*n* = 2–9) clusters and their ions. The energy gaps (*E*_*g*_, in eV), the electron affinities (EA, in eV), and ionization potentials (IP, in eV) of neutral Ni_2_Al_*n*_ (*n* = 2–9) clusters.

Clusters	Symmetries	*M* (in *μ*_B_)			BE (in eV)			*E*_*g*_ (in eV)	EA (in eV)	IP (in eV)
		Anion	Neutral	Cation	Anion	Neutral	Cation			
Ni_2_Al	C_2v_	2	1	2	8.70	7.19	−0.32	0.51	1.51	6.87
Ni_2_Al_2_	C_2v_	1	0	1	12.79	10.90	4.14	0.66	1.89	6.76
Ni_2_Al_3_	C_2v_	0	1	0	15.87	14.48	8.60	0.54	1.39	5.88
Ni_2_Al_4_	C_s_	1	0	1	18.41	16.84	10.76	0.86	1.57	6.08
Ni_2_Al_5_a	C_1_	0	1	0	21.58	19.61	13.73	0.41	1.97	5.88
Ni_2_Al_6_	C_2v_	1	2	1	25.40	23.05	16.69	0.33	2.35	6.36
Ni_2_Al_7_	C_2v_	0	1	0	28.14	26.07	20.27	0.43	2.07	5.80
Ni_2_Al_8_	C_1_	1	0	1	30.65	28.50	22.50	0.65	2.15	6.00
Ni_2_Al_9_	C_2v_	0	1	0	33.74	31.37	25.39	0.44	2.37	5.98

**Table 3:** The symmetries, the total magnetic moments (*M*, in *μ*_B_), and the binding energies (BE, in eV) for Ni_3_Al_*n*_ (*n* = 2–9) clusters and their ions. The energy gaps (*E*_*g*_, in eV) and the electron affinities (EA).

Clusters	Symmetries	*M* (in *μ*_B_)			BE (in eV)			*E*_*g*_ (in eV)	EA (in eV)	IP (in eV)
		Anion	Neutral	Cation	Anion	Neutral	Cation			
Ni_3_Al	C_2v_	2	0	2	12.35	10.89	4.32	0.47	1.46	6.57
Ni_3_Al_2_	C_2v_	1	0	1	16.67	14.99	8.45	0.62	1.68	6.55
Ni_3_Al_3_	C_s_	0	1	0	19.83	18.32	12.42	0.50	1.51	5.90
Ni_3_Al_4_	C_2v_	1	0	1	23.26	21.71	15.10	1.27	1.55	6.61
Ni_3_Al_5_	C_2v_	2	1	0	26.44	24.53	18.91	0.22	1.91	5.62
Ni_3_Al_6_	C_2v_	1	0	1	30.71	28.39	22.04	0.41	2.32	6.35
Ni_3_Al_7_	C_s_	0	1	0	33.30	31.07	25.15	0.29	2.23	5.92
Ni_3_Al_8_a	C_1_	1	0	1	35.62	33.43	27.71	0.48	2.19	5.72
Ni_3_Al_9_a	C_1_	0	1	0	38.71	36.22	30.32	0.29	2.49	5.90
